# Bacterial Antagonistic Species of the Pathogenic Genus *Legionella* Isolated from Cooling Tower

**DOI:** 10.3390/microorganisms10020392

**Published:** 2022-02-08

**Authors:** Kiran Paranjape, Simon Lévesque, Sébastien P. Faucher

**Affiliations:** 1 Department of Natural Resource Sciences, McGill University, 21 111 Lakeshore Drive, Ste-Anne-de-Bellevue, QC H9X 3V9, Canada; sebastien.faucher2@mcgill.ca; 2 Department of Medical Biochemistry and Microbiology, Husargatan 3, BMC, Uppsala University, 752 37 Uppsala, Sweden; 3 Département de Microbiologie et Infectiologie, Université de Sherbrooke, 3201 Jean Mignault, Sherbrooke, QC J1E 4K8, Canada; Simon.Levesque@USherbrooke.ca; 4 Service de Microbiologie, CIUSSS de l’Estrie-CHUS, 3001, 12è Avenue Nord, Sherbrooke, QC J1H 5N4, Canada

**Keywords:** *Legionella*, antimicrobials, cooling towers, whole genome sequencing

## Abstract

*Legionella pneumophila* is the causative agent of Legionnaires’ disease, a severe pneumonia. Cooling towers are a major source of large outbreaks of the disease. The growth of *L. pneumophila* in these habitats is influenced by the resident microbiota. Consequently, the aim of this study was to isolate and characterize bacterial species from cooling towers capable of inhibiting several strains of *L. pneumophila* and one strain of *L. quinlivanii*. Two cooling towers were sampled to isolate inhibiting bacterial species. Seven inhibitory isolates were isolated, through serial dilution plating and streaking on agar plates, belonging to seven distinct species. The genomes of these isolates were sequenced to identify potential genetic elements that could explain the inhibitory effect. The results showed that the bacterial isolates were taxonomically diverse and that one of the isolates may be a novel species. Genome analysis showed a high diversity of antimicrobial gene products identified in the genomes of the bacterial isolates. Finally, testing different strains of *Legionella* demonstrated varying degrees of susceptibility to the antimicrobial activity of the antagonistic species. This may be due to genetic variability between the *Legionella* strains. The results demonstrate that though cooling towers are breeding grounds for *L. pneumophila*, the bacteria must contend with various antagonistic species. Potentially, these species could be used to create an inhospitable environment for *L. pneumophila*, and thus decrease the probability of outbreaks occurring.

## 1. Introduction

*Legionella pneumophila* is the causative agent of Legionnaires’ disease, a severe and potentially fatal pneumonia. This organism is an aquatic bacterium ubiquitously found in engineered water systems (EWS) where it can colonize, survive, and grow. Examples of EWS that are known sources of *L. pneumophila* include water distribution systems, cooling towers, water reservoirs, misters, shower heads, and water faucets [1]. These systems usually produce aerosols that can be inhaled by people in their vicinity. If contaminated with *L. pneumophila*, inhaled aerosols can lead to the dissemination and colonization of the bacteria in the lungs, resulting in an atypical pneumonia known as Legionnaires’ disease. *L. pneumophila* is one of the more significant causes of waterborne diseases in developed countries [2,3]. The number of cases of Legionnaires’ disease has been on the rise in recent years. For instance, the United States reported an increase of more than fivefold in incidences from 2000 to 2017, and a 1.5-fold increase from 2013 to 2017 was observed in the European Union [4,5]. The number of cases of Legionnaires’ disease is believed to be underreported due to the lack of a common definition of the disease, efficient diagnostic tests, and surveillance programs in many countries or states [6]. Consequently, the actual number of cases is likely much higher. Mortality from Legionnaires’ disease varies amongst outbreaks but is usually around 8% to 12% [5,6]. This variability in mortality is due to the prevalence of specific risk factors associated with the disease in the affected population [6,7]. For example, a history of lung disease, smoking, old age (>50 years), immunosuppression, alcoholism, and cancer have all been linked with higher levels of mortality of Legionnaires’ disease [7]. In fact, hospital settings and assisted or senior living homes are common grounds for *Legionella* outbreaks. Consequently, different countries and organizations have implemented laws, guidelines, or standards to reduce the risk related to Legionnaires’ disease [8,9,10].

Moreover, *L. pneumophila* is divided into several sequence base types or SBTs based on allelic variants of marker genes [11,12]. The prevalence of these SBTs can vary depending on several factors. For example, around the world SBT1 is usually associated with environmental samples and sporadic cases, but rarely associated with large outbreaks [13]. This is potentially due to lowered virulence in this SBT than other ones. Added to this, different species of *Legionella* are also pathogenic. Around 10% of cases of Legionnaires’ disease are caused by other species, such as *L. longbeacheae*, *L. anisa*, and many others [13]. Recently, *L. quinlivanii*, a *Legionella* species discovered in the late 1980s, was reported in a human host, which was previously never observed [14,15]. Thus, different strains and species of *Legionella* are also of concern.

As *L. pneumophila* is ubiquitously found in EWS, understanding its ecology and the parameters that lead to its proliferation in these environments is important in order to better control or prevent outbreaks. Several biotic factors are known to affect positively or negatively *L. pneumophila*’s survival and growth. First, the bacterium is an intracellular parasite of various phagotrophic protozoan species, such as free-living amoeba and ciliates, and requires these host cells for growth in nature [16]. As a result, protist grazers are a major reservoir and disseminator of the bacterium in EWS [17]. Secondly, the presence or absence of *L. pneumophila* in EWS can be caused by certain bacterial species [18,19,20]. Several species inhibit the growth of *L. pneumophila* when tested by agar diffusion assays or in microcosm experiments. For instance, some *Staphylococcus* and *Bacillus* species inhibit *L. pneumophila* on agar plates through the production of hemolysins, bacteriocins, and surfactants [21,22]. Certain species can also inhibit the growth of host species. For instance, *Pseudomonas aeruginosa* is known to kill *Acanthamoeba castellanii* using its type 3 secretion system to inject effector proteins causing necrosis and apoptosis of the amoeba [23,24]. In another example, *Pseudomonas alcaliphila* has been shown to inhibit *L. pneumophila* and *Vermamoeba vermiformis* (host species) by secreting toxoflavin in biofilms [25]. On the other hand, certain *Cyanobacteria*, *Flavobacterium*, and *Brevundimonas* species promote the growth of *L. pneumophila* [19,26,27]. It is important to note that all of these interactions between different species may occur in the bulk water but are most likely to occur in biofilms, present in different water systems, as these environments are believed to be the major stronghold of *L. pneumophila* [28]. Consequently, environments containing *Legionella* species are potential sources for bacterial antagonists or mutualists. These bacterial species may be of interest, as they may represent potential biomarkers or control agents for *Legionella* spp., however, more work must be undertaken to characterize these species.

In the case of EWS, two studies in particular have shown that a high number of anti-*Legionella* bacterial species can be isolated from these environments [29,30]. Anti-*Legionella* species are species capable of inhibiting the growth of *Legionella* species, either through bactericidal, bacteriostatic, or competition interactions. These interactions can be specific at different taxonomic levels (strain, species, genus, etc.). Indeed, Guerrieri et al. found that 55 out of 80 bacterial isolates from tap water showed anti-*Legionella* activity [29]. Most of these isolates were species or strains of *Pseudomonas* but *Stenotrophomonas maltophila*, *Aeromonas hydrophyla*, *Burkholderia cepacia*, *Alacaligenes faecalis*, *Acinetobacter* spp., and *Flavobacterium* spp., were also shown to inhibit *L. pneumophila* [29]. On the other hand, Corre et al. tested 273 bacterial strains isolated from different water environments, including swimming pools, ponds, rivers, wells, and drinking water, and demonstrated that 178 of them had anti-*Legionella* activity [30]. Their findings also demonstrated that a high diversity of waterborne bacteria, mainly from the *Gammaproteobacteria* and *Firmicute* groups, can have antimicrobial activity against *L. pneumophila*. The latter two phyla are known to produce a lot of secondary metabolites and antimicrobial peptides. This suggests that there may be a large pool of different antimicrobials that have yet to be characterized in these environments.

So far, the presence of antagonistic bacterial species towards *L. pneumophila* in cooling towers has not been studied. We have previously analyzed the bacterial community of 18 cooling towers and demonstrated that several bacterial taxa were negatively correlated with *Legionella* [20]. Higher levels of these bacterial taxa were associated with lower levels of *Legionella* spp. Therefore, our goals were to isolate bacterial species from these cooling towers and test their antimicrobial activity towards *L. pneumophila* and *Legionella quinlivanii*. The second goal was to determine the potential genetic elements that could cause the inhibition through whole genome sequencing.

## 2. Materials and Methods

### 2.1. Legionella pneumophila Strains

Table 1 shows the characteristics of the *L. pneumophila* and *L. quinlivanii* strains used in this study. The strains are characterized by a strain number, a strain name, the species, the sequence base type (SBT), and the environment from where they were isolated. Moreover, ATCC33152 was isolated during the first outbreak of Legionnaires’ disease in Philadelphia in 1976, and ID120292 caused the 2012 outbreak in Quebec City, Canada [31]. The other strains were either isolated from the environment (E) or from patients (P) and were obtained from the Laboratoire de santé publique du Québec (LSPQ), Canada. Patient strains were either outbreak strains or sporadic human cases and environmental strains were obtained from contaminated cooling towers typed in a previous study [32]. The *L. quinlivanii* strain was obtained from a bronchoalveolar lavage specimen from a patient [15]. The strains were grown on ACES-buffered CYE (Charcoal Yeast Extract) plates (yeast extract 10 g, ACES buffer 10 g, activated charcoal 2.0 g, L-cysteine 0.4 g, ferric pyrophosphate 0.25 g in 1 L of water, pH 6.90) at 30 °C for 4 days.

### 2.2. Isolation of Inhibitory Bacterial Strains of Legionella pneumophila

Cooling tower water samples were examined for their potential to contain bacterial species that could inhibit *L. pneumophila* on plate. Briefly, water was collected in sterile 1 L bottles from the basins of two cooling towers in Montreal, Canada, and one model cooling tower built in our lab [33]. The water samples were vigorously shaken and serially diluted in filter sterilized sample water, to a dilution of 10^−6^. CYE agar plates were layered with 5 mL of soft agar (0.5% agar in distilled water), inoculated with 100 µL of 0.2 OD_600nm_ of *L. pneumophila* suspension in AYE (Yeast extract 10 g, ACES buffer 10 g, L-cysteine 0.4 g, ferric pyrophosphate 0.25 g in 1 L of distilled water, pH 6.90). CYE medium was chosen as it is the only agar medium known to support growth of *Legionella* species. The soft agar was let to solidify for 15 to 30 min in a biological safety cabinet. The dilutions were spread on the CYE agar by gently flooding 1 mL of solution on to the soft agar layer. The dilution was spread by gently shaking and tilting the agar plate, after which the excess liquid was aspirated with a pipette. The plates were left to dry for 30 min in a biological safety cabinet and then incubated at 30 °C for 4 days. The inhibiting colonies could be visualized by the formation of an inhibition zone on the *L. pneumophila* lawn. These colonies were re-streaked three times on CYE plates to obtain pure cultures. Stock cultures of these isolates were made in 15% glycerol in AYE medium.

### 2.3. Identification of Bacterial Isolates

Bacterial isolates were first identified by sequencing their *16S rRNA* gene. Briefly, bacterial DNA was extracted by lysing a single colony in 25 µL of 0.5 NaOH. The suspension was incubated at room temperature for 10 min and then neutralized with 25 µL of 1 M tris-HCL, pH 7.5 and diluted with 450 µL of sterile distilled water. The *16S rRNA* gene was amplified by PCR using the bacterial primers 27F (5′-AGAGTTTGATCMTGGCTCAG-3′) and 1492R (5′-TACGGYTACCTTGTTACGACTT-3′). The PCR product was then sent for Sanger sequencing at the Plateforme Génomique de l’Université Laval, Canada. The sequences were then compared to the NCBI nucleotide database using BLAST [34].

### 2.4. Testing Inhibition of Isolates with Different Legionella Strains

We further tested the isolates’ antimicrobial activity towards eight different strains of *L. pneumophila* and one strain of *L. quinlivanii*. The *Legionella* strains were inoculated on CYE agar using the soft-agar approach described above. Pure cultures of the inhibiting isolates were suspended in AYE at 0.2 OD_600nm_ and 10 µL was spotted in the centre of the agar plates. The spots were left to dry for 15 to 30 minutes and the plates were then incubated at 30 °C for 4 days. After incubation, the inhibition zone diameters were measured to compare antimicrobial activity and compare susceptibility levels between *Legionella* strains.

### 2.5. Whole Genome Sequencing of Anti-Legionella Isolates

Genomic DNA was extracted from the isolates using the Wizard genomic DNA purification kit (Promega, Madison, WI, USA). The genomic DNA quality was verified on a 0.8% agarose gel and the quantity was measured using the Quant-iT PicoGreen dsDNA assay kit (Thermofisher, Waltham, MA, USA). The DNA library for whole genome sequencing was prepared using the Nextera XT DNA library prep kit (Illumina, San Diego, CA, USA). The manufacturer’s instructions were followed. The library was run on an Agilent Technology 2100 Bionalyzer (Agilent, Santa Clara, CA, USA) to evaluate proper DNA fragment size. After evaluation of proper fragmentation, the library was manually normalized to 2 nM and then pooled together. The pooled library was denatured with 0.2 N (normality) NaOH and incubated for 5 minutes at room temperature. The solution was neutralized with 200 mM of Tris-HCl (pH 7.0). The denatured library was diluted to 20 pM with HT1 buffer and diluted again to a loading concentration of 12 pM. PhiX was diluted to 4 nM in HT1 buffer and denatured with 0.2 N NaOH at room temperature for 5 min. The denatured PhiX was then diluted to 20 pM with HT1 buffer (Illumina, San Diego, CA, USA). The denatured library was spiked-in at 1% with PhiX control. The solution (600 µL) was loaded into a MiSeq Reagent kit V3 (600 cycles) and sequenced on a MiSeq platform (Illumina, San Diego, CA, USA). The raw reads have been uploaded to NCBI’s Sequence Read Archive (SRA) under the bioproject accession number PRJNA787617.

The read quality was evaluated using FastQC [35]. The forward and reverse sequences were removed using Trimmomatic (v0.39) with the following commands: LEADING: 10 TRAILING: 10 SLIDINGWINDOW: 5: 20 MINLEN: 36 [36]. The forward and reverse reads were assembled de novo using Spades (v3.13) for each isolate [37]. The reads were first corrected using the “only-error-correction” option and assembled using the “only-assembler” option. When assembling the reads, the k-mer length was set to 21, 33, 55, 77, 99, and 127. The assembled genomes were uploaded to MiGA (Microbial Genome Atlas, v0.3.12) server, and the NCBI Prok module was used to identify the taxonomy and novelty of the isolate [38]. Of note, when analyzing with MIGA, one of the bacterial isolates, SPF474, was observed to have a high percentage of contamination in its genome, but phylogenetic analysis was still able to identify it at a high percentage level and with high confidence. Thus, contaminating reads were removed from SPF474. This was done by mapping all reads to the genome of *Bacillus amyloliquefaciens* IT-45 (RefSeq NC_020272.1 from NCBI) using BWA-mem and Samtools [39,40]. The unmapped reads were removed, and the mapped reads were *de novo* assembled using Spades (v3.13). This new assembly was uploaded to MiGA for phylogenetic analysis. The *B. amyloliquefaciens* IT-45 strain was used as it is considered a representative genome according RefSeq and sanger sequencing with the MiGA results indicated that SPF474 was highly related to *Bacillus amyloliquefaciens*. The assembled genomes were also uploaded to Antismash (v5.0) in order to identify, annotate, and analyze secondary metabolite biosynthesis gene clusters, using the relaxed detection strictness parameter [41]. The ClusterBlast, KnownClusterBlast, and SubClusterBlast options were used to evaluate homology with known antimicrobial sequences.

## 3. Results

### 3.1. Inhibition Assay

**Figure 1:**
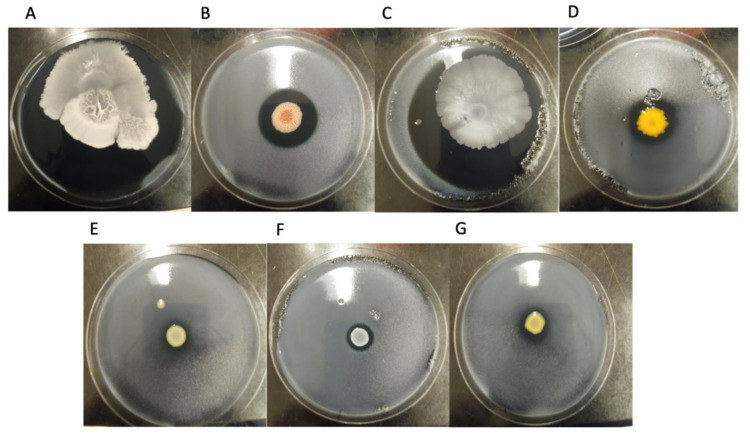
Isolates showing inhibition zones on a lawn of *L. pneumophila* (LpS256P) on CYE agar incubated at 30 °C for 4 days. Isolates are: (**A**) *Bacillus amyloliquefaciens* (SPF474), (**B**) *Bacillus paralicheniformis* (SPF497), (**C**) *Bacillus subtilis* (SPF437), (**D**) *Chryseobacterium* sp. (SPF475), (**E**) *Cupriviadus* sp. (SPF499), (**F**) *Staphylococcus epidermidis* (SPF476), and (**G**) *Stenotrophomonas* sp. (SPF498), identified by *16S rRNA* and whole genome sequencing.

**Figure 2:**
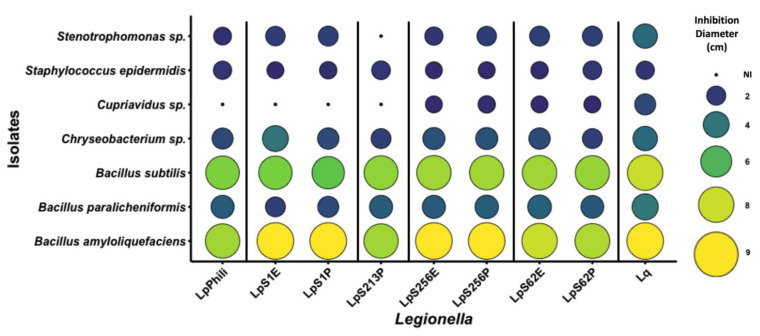
Inhibition zone diameters, in centimeter, produced by bacterial isolates on lawns of different strains of *Legionella* grown on CYE agar incubated at 30 °C for 4 days. The inhibition zone diameter is indicated by the size of the circle and by colour scale (from purple to yellow; NI: no inhibition).

Seven bacterial colonies inhibiting *L. pneumophila* growth on plates were isolated from cooling towers and a model cooling tower. Their ability to inhibit different SBTs of *L. pneumophila* and *L. quinlivanii* was tested. Figure 1 shows photographic examples of the inhibition assay when testing with *L. pneumophila* strain LpS256P. Figure 2 shows the diameter of the inhibition zone created by each bacterial isolate for each strain of *Legionella* tested.

As expected, the inhibition of *L. pneumophila* varied according to the bacterial isolate tested. For instance, *B. amyloliquefaciens* and *B. subtilis* created large inhibition zones ranging from 7 cm to 9 cm (total inhibition). On the other hand, *Chryseobacterium* sp. and *B. paralicheniformis* both created intermediate inhibition zones, between 2.5 cm and 4 cm in diameter. The other isolates (*Cupriavidus* sp., *S. epidermidis*, and *Stenotrophomonas* sp.) created small inhibition of 2 cm or smaller. The results also suggest that the susceptibility to the anti-*Legionella* bacterial isolates varied according to the SBT and the source from which the *L. pneumophila* strain was isolated from, i.e., patient or environment. Thus, the variation with SBT was most observable with the *Cupriavidus* sp. isolate, which inhibited around half of the *L. pneumophila* strain tested. As a result, SBT37 (LpPhili), SBT1 (LpS1E and P), and SBT213 (LpS213P) had no susceptibility to *Cupriavidus* sp., whereas SBT256 (LpS256E and LpS256P) and SBT62 (LpS62E and LpS62P) were susceptible, creating around 2 cm inhibition zones. In another example, *B. paralicheniformis* created an inhibition zone of between 2.2 to 2.5 cm for SBT1 (LpS1E and P), but the inhibition was between 3.1 to 3.3 cm for the other *L. pneumophila* strains. Interestingly, *Chryseobacterium* sp. created different sized inhibition zones for LpS1E and LpS1P. In this case, the environmental strain was less susceptible, creating a 2.6 cm inhibition zone, than the patient isolated strain, which created a 4 cm zone. The *L. quinlivanii* strain had larger inhibition diameters than the *L. pneumophila* strains, for most of the bacterial isolates tested. This suggests that different strains within the same SBT may have variability in susceptibility to certain antimicrobials. Though the strains of *L. pneumophila* and *L. quinlivanii* tested did not come from the cooling towers sampled, an interesting experiment would be to evaluate if these same cooling towers had a *Legionella* population and assess their susceptibility levels to the anti-*Legionella* bacterial isolates to determine if the laboratory findings have a role in the real world.

### 3.2. Taxonomic Classification of Bacterial Isolates

After sequencing and assembly of the anti-*Legionella* bacterial isolates, taxonomy was inferred using the Microbial Genome Atlas [38]. Table 2 shows the results from this analysis. The taxonomic classification of three of the isolates could be identified with a high level of confidence. Indeed, SPF437, SPF476, SPF497 had average nucleotide identities (ANI) above 99.5% at *p*-values below 0.01. The *p*-value indicates the probability of our query genome being wrongly classified with the reference genome from NCBI’s RefSeq database [38]. Consequently, SPF437, SPF476, SPF497 were respectively classified as *Bacillus subtilis*, *Staphylococcus epidermidis*, and *Bacillus paralicheniformis*.

As mentioned previously, SPF474 had some level of contamination in the whole genome sequencing results according to MiGA, however, the ANI was at 99.91% with *Bacillus amyloliquefaciens* LL3NC017190 with a *p*-value of 8.02 × 10^−5^. After clean-up of the genome, the MiGA results showed that SPF474 had a 99.98% ANI with *B. amyloliquefaciens* LL3NC017190 at a *p*-value 8.02 × 10^−5^, and very low contamination levels. Furthermore, the additional sequencing of its *16S rRNA* gene also indicated that SPF474 was related to *Bacillus amyloliquefaciens*. Indeed, the reverse and forward sequence aligned to *B. amyloliquefaciens* strain RESI-50 (Accession: MT542326.1; e-value: 0.0; percent identity: 99.88%; query cover: 100%) and *B. amyloliquefaciens* strain 3820 (Accession: MT538668.1; e-value: 0.0; percent identity: 99.62%; query cover: 100%), respectively. Consequently, the results suggest that the contamination had little to no effect in identifying the isolate. Though not shown here, when inputting the genome in Antismash before clean-up, several sequences detected in its genome were similar to gene sequences found in *Legionella* species. Thus, this may indicate that the foreign sequences came from a cross-contamination event during one of the preparation steps for whole genome sequencing.

The taxonomic classification of SPF498, SPF499, and SPF475 was less confident, as their ANI percentage varied between 86% and 95% at *p*-values sometimes above 0.05. Indeed, there is a high probability that SPF475 and SPF498 belong to the genera ascribed, as their *p*-values were below 0.01 when comparing at the genus level (SPF498 *p*-value = 0.0063 for *Stenotrophomonas*; SPF475 *p*-value = 0.0085 for *Chryseobacterium*), but they probably belong to species not represented in the database. On the other hand, SPF499 had an ANI of 86.84% with *Cupriavidus pauculus* NZCP033969 at *p*-values of 0.468 and 0.0338 at the species level and the genus level, respectively. Though still debatable, the species boundaries using ANI percentage is usually set at a cut-off of 96% or higher [42]. The ANI being smaller than this cut-off suggests that SPF499 might be a new species, and even a new genus, depending on the cut-off used for the ANI% result and the *p*-value.

### 3.3. Identification of Putative Secondary Metabolites

In order to identify putative antimicrobial compounds produced by the different bacterial isolates, the assembled genomes were analyzed using AntiSMASH server (Antibiotics and Secondary Metabolite Analysis Shell) [41]. This tool allows the identification and analysis of biosynthetic gene clusters (BGCs) within bacterial genomes. Some of these BGCs may allow the production of antimicrobial compounds, such as antibiotics or bacteriocins [41]. AntiSMASH will also BLAST the identified clusters to known antimicrobial sequence databases. Table 3 represents a summary of the results obtained from AntiSMASH for the different bacterial isolates. The BGCs identified were categorized by their similarity percentage to known BGCs in the database. Thus, BGCs with more than 70% were categorized in the high similarity group, BGCs with less than 70% similarity were categorized in the low similarity group, and BGCs with 0% similarity were categorized as unassigned.

Overall, the genomes were found to contain several BGCs. The numbers in each isolate varied greatly from 3 to 16 BGCs. The *B. paralicheniformis* and *B. subtilis* isolates had the most BGCs and some were highly homologous to known antimicrobials, such as bacitracin or fengycin. On the other hand, *Cupriavidus* sp. (SPF499) and *Chryseobacterium* sp. (SPF475) also possessed high numbers of BGCs, but these BGCs had low similarity levels to any of the antimicrobials gene products in the database. AntiSMASH only detected three BGCs for the *Stenotrophomonas* (SPF498) and *Staphylococcus* (SPF436) isolates.

**Figure 3:**
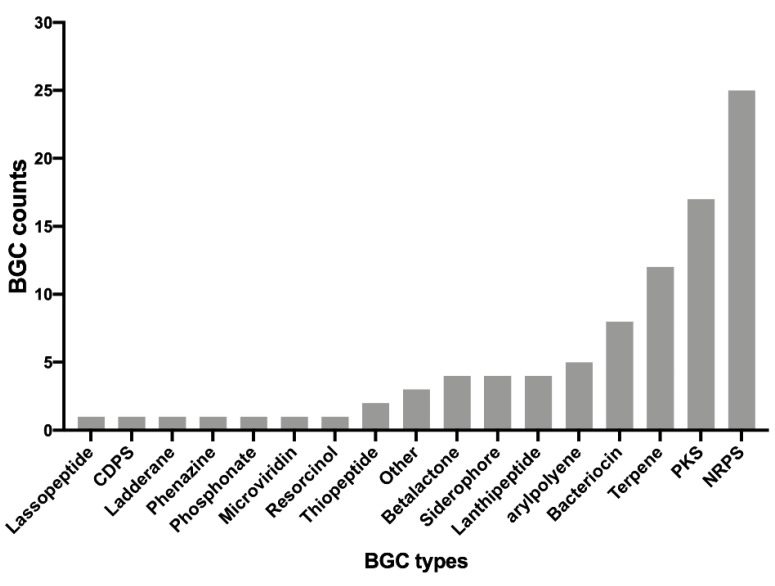
Diversity of BGC types identified in the genomes of the bacterial isolates using AntiSMASH.

We examined the diversity of the BGCs present throughout the different genomes by counting the total sum of each type of BGC identified. The results showed that a total of 17 different types of BGCs could be identified. These can be visualized in Figure 3. Unsurprisingly, non-ribosomal peptides synthetase (NRPS) were the most abundant antimicrobial clusters found in the different genomes. NRPSs have a wide range of biological activity and are known to produce several antibiotics, such as penicillin or cephalosporins [43]. Polyketide synthase (PKS), terpenes, and bacteriocins were the next most abundant BGCs. Finally, the rest of the BGCs were found at abundance levels of less than 5 counts, and 7 BGCs (Lassopeptide, CDPS, Ladderane, phenazine, phosphonate, microviridin, and resorcinol) were only counted once.

## 4. Discussion

In this study, we isolated and characterized seven bacterial species from cooling tower water samples capable of inhibiting *L. pneumophila* and *L. quinlivanii* on CYE plates. Their genomes were sequenced to get a better understanding of the potential antimicrobials that could be produced, and the genes associated with these antimicrobials. So far, research has shown that a wide variety of organisms from EWS can inhibit *L. pneumophila*. Research has not looked into antagonistic species of *L. quinlivanii*. Our study shows that cooling towers can harbour anti *L. pneumophila* species and that these species can also inhibit other *Legionella* species (such as *L. quinlivanii*), indicating that these inhibitory strains could potentially be used against several *Legionella* pathogens.

The findings confirm that a wide variety of bacterial species can inhibit *L. pneumophila* in water systems. Indeed, *Firmicutes*, *Bacteroidetes,* and *Proteobacteria* were all identified in this study, with most species belonging to the *Firmicutes*. This is in agreement with Corre et al. [30]. It is important to note that since we only tested inhibition on CYE growth medium, the actual diversity of inhibitory organisms may be underrepresented. Indeed, the number of unculturable microorganisms is far higher than the number of culturable organisms [44,45]. In our case, the methodology created a bias for the selection of non-fastidious mesophiles due to the incubation at 30 °C on nutrient rich media. An alternative strategy would be to isolate microbes on different media and slowly acclimate them to CYE agar before doing the *Legionella*-inhibition assay. Alternatively, it may be more representative, but challenging, to evaluate *Legionella* survivability in co-infection models (host/*Legionella*/isolate), in lab grown biofilm experiments, or creating new growth media, other than CYE, and testing for inhibition at different growth temperatures. However, the isolation and identification of SPF499 suggests that novel and uncharacterized non-fastidious bacterial species can still be discovered.

The AntiSMASH results revealed that the bacterial isolates contained an array of BGCs potentially coding for a wide variety of antimicrobials. Over 17 different BGC types were uncovered, suggesting that *Legionella* spp. could be inhibited through diverse mechanisms. More specifically, the data suggests that the inhibition could be done through direct mechanisms. For instance, NRPS, PKS, terpenes, and bacteriocins were the most abundant antibacterial identified in the isolates. These compounds usually directly act on the bacterial cells, targeting specific elements and causing bactericidal or bacteriostatic effects [46,47,48,49]. On the other hand, the identification of several siderophore clusters could indicate that *L. pneumophila* may be inhibited indirectly, such as through competition for nutrients. For instance, staphyloferrin genes were identified in the *S. epidermidis* (SPF476) isolate. Staphyloferrin is a powerful siderophore used by *Staphylococcus* species and thus could prevent *L. pneumophila* from acquiring iron for growth [50]. The variety of BGCs identified could also indicate that a combination of different mechanisms could explain the inhibition of *L. pneumophila*. For instance, certain species may be able to out-compete *L. pneumophila* through production bactericidal agents, faster acquisition of nutrients, and faster growth rates. A potential follow-up experiment would be to knock-out the suspected bactericidal BGCs from the different isolates to assess if isolates are not using a variety of mechanisms to cause inhibition of *L. pneumophila*.

Some of the BGC sequences identified were previously shown to inhibit *L. pneumophila* on plate. For instance, Loiseau et al. showed that surfactin produced by *B. subtilis* can create an inhibition zone on a lawn of *L. pneumophila* [22]. However, our isolates of *B. subtilis* created a much larger inhibition zone. This could be due to methodological difference, such as the use of a soft agar layer in the present study allowing for better diffusion of the surfactin on the growth plate. It is also possible that our strain may produce other antimicrobials that work in synergy to inhibit *L. pneumophila* growth. As shown in Table 3, our *B. subtilis* isolate contains BGCs related to surfactin, bacilysin, and bacillibactin. Potentially these compounds could work in combination to inhibit *L. pneumophila*. Notably, bacilysin is an antibiotic, which works against a wide range of bacteria, and bacillibactin is a siderophore capable of chelating iron [51,52]. Of note, the *B. paralicheniformis* isolate was shown to contain a BGCs associated with the production of lichenysin, a lipopeptide surfactant almost identical to surfactin [22,53]. Pure lichenysin has antibacterial activity against several species, such as *Acinetobacter* sp., *Bacillus* sp., and *Pseudomonas* sp., but this has not been tested in *Legionella* species [53]. However, due to the high similarity of surfactin and lychenisin, it is of our opinion that this is most likely the compound that causes the inhibition of *L. pneumophila* on plate. Further, research is required to confirm this phenotype.

Several of the BGCs identified have very low similarity or did not relate to any known antimicrobial biosynthetic clusters from the MiBIG database used in AntiSMASH. For instance, the *Chryseobacterium* sp. (SPF475) contained several BGCs that were similar to desferrioxamine, flexirubin, and caratenoid, but at very low similarity levels (below 50%). Similarly, only low similarity BGCs were identified in *Stenotrophomonas* sp. Therefore, the *Legionella*-inhibition phenotype of these isolates could be due to novel compounds, suggesting that cooling towers are a rich source of novel and uncharacterized antimicrobial compounds that could potentially be used for clinical or industrial purposes.

In conclusion, several bacterial isolates that showed anti-*Legionella* activity were isolated from different cooling tower water samples. This study confirms that cooling towers can harbour a diversity of anti-*Legionella* bacterial species from different phyla. Whole genome sequencing revealed that several known antimicrobials were potentially causing the inhibition of *L. pneumophila* on plate; however, the number of uncharacterized antimicrobials was much greater, suggesting a potential pool of antimicrobials. As these inhibitory species of *L. pneumophila* could be used for industrial purposes, several interesting follow up studies could be pursued. First, evaluating the specificity of these antimicrobials within the *Legionella* genus, either focusing on different strains of *L. pneumophila* or for different species of *Legionella*, and to examine if these same antimicrobials can inhibit other pathogens would be of value. Some of our results indicate that there was variability with the different strains tested as well with *L. quinlivanii,* suggesting some specificity. Additional research is required to get a better overview. Furthermore, consideration should be focused on evaluating the pathogenicity of the isolates towards humans, as pathogenic species would probably cause unwanted consequences for downstream applications. Finally, more practical knowledge concerning how to directly use inhibitory species would be more beneficial for industrial purposes. Thus, questions such as evaluating the efficiency of these inhibitory isolates at reducing the *L. pneumophila* load in different water systems, gauging if seeding is better than tweaking physical chemical parameters of the water system so that these species can colonize naturally the water system, analyzing if these species are harmful to humans, or assessing if using directly the antimicrobial is cost effective along with routine biocides, are all important questions that would be interesting to pursue.

## Figures and Tables

**Table 1:** Information on the species, the sequence base type, and the source of the *Legionella* strains used in this study.

Strain Name *	Strain Number **	Species	Sequence Base Type (SBT)	Source
LpPhili	ATCC33152	*L. pneumophila philadelphia 1* (ATCC33152)	37	Patient
LpS62P	ID143016	*L. pneumophila*	62	Patient
LpS62E	ID120292	*L. pneumophila* (2012 Quebec City Outbreak)	62	Environmental
LpS1P	ID126851	*L. pneumophila*	1	Patient
LpS1E	ID142903	*L. pneumophila*	1	Environmental
LpS256P	ID128014	*L. pneumophila*	256	Patient
LpS256E	ID128471	*L. pneumophila*	256	Environmental
LpS213P	ID120882	*L. pneumophila*	213	Patient
Lq	ID143958	*L. quinlivanii*	NA	Patient

* For convenience, the strains were given a strain name based on species, sequence-type, and source. ** Strain numbers starting by ID were obtained from the LSPQ, Canada.

**Table 2:** Taxonomic classification of the anti-*Legionella* isolates.

Strain Number	Closest Species (% ANI)	*p*-Value at Species Level	*p*-Value at Genus Level	Predicted Number of Proteins	Genome Size (bp)	Source
SPF474	*Bacillus amyloliquefaciens* LL3 NC 017190 (99.98%)	8.02 × 10^−5^	8.02 × 10^−5^	3638	3,522,847	Cooling tower MTL3 [20]
SPF497	*Bacillus paralicheniformis* NZ CP033389 (100%)	8.02 × 10^−5^	8.02 × 10^−5^	4407	4,415,689	Cooling tower MTL3 [20]
SPF498	*Stenotrophomonas* sp. MYb57 NZ CP023271 (95.67%)	0.124	0.0063	4091	4,581,475	Cooling tower MTL5 [20]
SPF499	*Cupriviadus pauculus* NZ CP033969 (86.84%)	0.468	0.0338	6397	6,854,167	Cooling tower MTL3 [20]
SPF475	*Chryseobacterium indologenes* NZ CP018786 (90.65% ANI)	0.296	0.0085	4971	5,302,653	Cooling tower MTL5 [20]
SPF476	*Staphylococcus epidermidis* ATCC 12228 NC 004461 (99.59%)	0.0086	0.0014	2422	2,530,472	Cooling tower MTL3 [20]
SPF437	*Bacillus subtilis subsp inaquosorum* NZ_CP013984 (99.98% ANI)	8.02 × 10^−5^	8.02 × 10^−5^	4123	4,195,215	Cooling tower model [33]

**Table 3:** Biosynthetic gene clusters identified by AntiSMASH server in the different bacterial genomes.

Strain Name	Number of BGCs	High Similarity Clusters (>70% Similarity)	Low Similarity Clusters (<70% Similarity)	Number of Unassigned BGCs
*Bacillus amyloliquefaciens*	10	Bacillaene (100%), Bacillibactin (100%), Bacilysin (100%), Fengycin (93%)	Butirosin A/B (7%), Bacillomycin (60%), Surfactin (39%)	3
*Bacillus paralicheniformis*	14	Fengycin (73%), Lichenysin (100%), Bacitracin (88%),	Bacilibactin (53%), Fengycin (23%), Butirosin (7%), Haloduracin (40%), Fengycin (20%)	6
*Stenotrophomonas sp.*	3	0	Myxochelin (25%), APE Vf (35%)	1
*Cupriavidus sp.*	9	0	Desferrioxamine (50%), APEVf (40%), WS9326 (12%)	6
*Chryseobacterium sp.*	12	0	Desferrioxamine(50%), Flexirubin (52%), Flexirubin (22%), Caratenoid (28%)	8
*Staphylococcus epidermidis*	3	Staphyloferrin (100%)	0	2
*Bacillus subtilis*	16	Subtilosin A (100%), Bacilysin (100%), Surfactin (82%), Bacillibactin (100%), Fengycin (80%), Sublancin (100%),	Plipastatin (53%), Zwittermycin (18%), Aurantinins (21%), Aurantinins (39%), Plipastatin (23%), Aurantinins (28%)	4

## Data Availability

The raw MiSeq reads for each of the seven bacterial isolates have been uploaded to NCBI’s Sequence Read Archive (SRA). The Bioproject accession number is PRJNA787617.
